# Microevolution during Chronic Infection May Lead *T. asahii* to Coexist with the Host

**DOI:** 10.1155/2024/5518156

**Published:** 2024-07-30

**Authors:** Gen Ba, Xuelian Lv, Xin Yang, Wenling Wang, Junhong Ao, Rongya Yang

**Affiliations:** ^1^ Department of Pathology The 305 Hospital of PLA, Beijing, China; ^2^ Department of Dermatology Beijing An Zhen Hospital, Beijing, China; ^3^ Department of Dermatology The Seventh Medical Center of PLA General Hospital, Beijing, China

## Abstract

**Background:**

*Trichosporon asahii* (*T. asahii*) is part of the cutaneous fungal microbiota in humans and can cause lethal opportunistic infection. During infection, microorganisms can adapt to their environment by adjusting gene expression and cellular activities.

**Objectives:**

Investigation of the microevolutionary changes in *T. asahii* during chronic infection.

**Methods:**

Two *T. asahii* strains were isolated from a chronic trichosporonosis patient between a 15-year interval, and the microevolutionary changes were compared by the immune response of dendritic cell (DC), mice survival model, and transcriptome sequencing analysis.

**Results:**

Compared with the primary *T. asahii* strain, the microevolved strain induced much lower expression of TNF-*α* by mice bone marrow-derived DC and had a much superior survival rate, a total of 2212 significantly differentially expressed genes were identified in the microevolved strain, and functional analysis showed significance in the downregulated transcription and metabolic process, especially the valine, leucine, and isoleucine degradation pathways, which were associated with pathogenicity and virulence; hence, the results were highly consistent with the decreased immunogenicity and virulence of the microevolved strain.

**Conclusions:**

These results demonstrated that the microevolution during chronic infection could induce changes in immunogenicity, virulence, and transcriptome, which might lead *T. asahii* to coexist with the host.

## 1. Background

*Trichosporon asahii* (*T. asahii*) is part of the cutaneous fungal microbiota in humans and can cause lethal opportunistic infection in immunocompromised patients [1]; however, there are increasing cases of *T. asahii* infection in immunocompetent hosts [2–4]. During infection, microorganisms can adapt to changes in their environment by adjusting gene expression and cellular activities [5], such microevolution can be illustrated by biofilm development in *Candida albican* through evolved transcriptional network controls [6] and hyphal formation of *Candida albican* to escape from phagocytosis [7].

As the most powerful antigen-presenting cell (APC), dendritic cell (DC) can motivate and regulate immune responses against fungi: phagocyte fungi and present fungal antigen, to mediate innate and adaptive immunity, at the same time release proinflammatory cytokines such as TNF-*α* [8, 9].

We previously obtained two *T. asahii* strains from facial skin lesions of a chronic trichosporonosis patient in 2000 (called AS2.2174) and 2015 (called BM1403), the 26S rDNA D1/*D*2 region of AS2.2174 was sequenced and confirmed to be *Trichosporon asahii* strain, and BM1403 was 100% mapped onto AS2.2174 genome and morphologically and biochemically same with AS2.2174. Thus, AS2.2174 was the ancestor of BM1403 [10, 11]. The patient displayed no evidence of underlying disease during infection, which reminded us of microevolution of *T. asahii* during chronic infection. Moreover, few works have focused on the interactions between DC or mice model and *T. asahii* undergoing microevolution. Hence, in this study, we compared the microevolutionary changes in *T. asahii* by the immune response of DC, mice survival model, and transcriptome sequencing analysis, and our results showed that *T. asahii* may perform microevolution to coexists with the host through immunogenicity, virulence, and transcriptome adaption.

## 2. Materials and Methods

### 2.1. Strains and Growth Condition

The two *T. asahii* strains, AS2.2174 and BM1403 samples, were both stored in 20% glycerol at −80°C until use, and then, they were transferred into fresh yeast extract peptone dextrose (YPD) liquid medium in a shaker incubator and cultured at 150 rpm at 35°C for 18 h. After that, 20 ml of each *T. asahii* strain was centrifuged at 3000 rpm at 4°C for 10 min, then washed with normal saline, and resuspended in normal saline at 1 × 10^4^ CFU/ml density. 5 *μ*l of each suspension was spotted onto potato dextrose agar (PDA) plates at 35°C for 7 days.

### 2.2. Bone Marrow-Derived Dendritic Cell (BMDC) Culture

Bone marrow cells were collected from the femur and tibia bones of C57BL/6 mice, erythrocyte lysate was added to remove red blood cells, and the supernatant was discarded after centrifugation (1500 r/min, 5 min). Cells were resuspended by RPMI 1640 medium with 10% fetal bovine serum and plated at a concentration of 2 × 10^6^ cells well in 6-well culture plates, supplemented with GM-CSF (20 ng/ml, PeproTech, USA) and IL-4 (10 ng/ml, PeproTech, USA) in a cell incubator (37°C, 5% CO_2_). Half of the medium was changed, and cytokines were supplemented every other day. On day 8, suspended and semisuspended cells were harvested in new 6-well plates at the same concentration for coincubation with *T. asahii* strains.

### 2.3. Strains and BMDC Coincubation

The two strains of *T. asahii* cultured on YPD as described above were washed and resuspended with normal saline and took half for heat inactivate (65°C, 3 h). Then, the activated and inactivated *T. asahii* strains were coincubated with BMDC in each well (BMDC: *T. asahii* = 1: 5) in a cell incubator (37°C, 5% CO_2_) for 24 h.

### 2.4. Cytokines mRNA of BMDC Expression by Real-Time PCR

Supernatants of coincubation were collected and centrifuged (2500 r/min, 10 min). The total RNA was extracted with TRIzol reagent (Invitrogen, USA) and the cDNA using PrimeScript RT Master Mix (Takara, Japan). Real-time PCR and the 2^-∆∆CT^ method were used to detect TNF-*α*, which represents the immune response of BMDC to *T. asahii* strains. The primer sequences of TNF-*α*: forward: 5′-GGAACACGTCGTGGGATAATG-3′, reverse: 5′-GGCAGACTTTGGATGCTTCTT-3′; GAPDH: forward: 5′-AATGGATTTGGACGCATTGGT-3′, reverse: 5′-TTTGCACTGGTACGTGTTGAT-3′. The amplification system includes 2 × PCR mix (Invitrogen, USA) 10 *µ*l, forward primer (5′–3′) 0.5 *µ*l, reverse primer (3′–5′) 0.5 *µ*l, cDNA 4 *µ*l, and dH_2_O 5 *µ*l make up to 20 *µ*l. Each experiment was repeated in triplicate.

### 2.5. Murine Survival Study

C57BL/6 mice (male 6–8 week, 18 to 20 g) were obtained from the Tsinghua Laboratory Animal Resources Center (Beijing, China) and housed in standard cages with ad libitum access to water and food. The two strains of *T. asahii* cultured in YPD as described above were washed and resuspended with normal saline at 1 × 10^7^ CFU/ml density and were injected into the lateral tail vein at a dose of 0.1 mL per mouse, 9 received AS2.2174, and 10 received BM1403. All mice surviving to day 16 were humanely sacrificed.

### 2.6. RNA Extraction, Library Construction, and Sequencing

The two strains of *T. asahii* cultured on PDA as described above were used for RNA isolation. The total RNA was extracted with TRIzol reagent (Invitrogen, USA), the purity was analysed by NanoDrop 2000 (Thermo Fisher Scientific, USA), and the integrity was evaluated by Agilent 2100 (Agilent Technologies, USA). Then, mRNA was enriched by magnetic beads with Oligo (dT, Dynabeads, Norway) from the qualified total RNA. cDNA libraries were constructed by mRNA templates and purified by AMPure XP beads (Beckman, USA).

### 2.7. Transcriptome Statistics

The N-containing, low quality, adapter-related reads were removed after high-throughput sequencing, leaving the clean reads. Then, the transcriptome reads of the two strains were mapped with the *T. asahii* genome (CBS 2479 strain) using the TopHat2 software. Gene abundance levels were evaluated by fragments per kilobase of exon model (FPKM) per million mapped reads.

### 2.8. Expression Level Analysis

HTSeq software was used for expression level analysis, the threshold of FPKM was >1, and the gene expressions between two strains were compared by the log_10_(FPKM + 1) value. DEGseq software was used for differentially expressed genes.

DEGseq software was used for differentially expressed genes (DEGs) analysis, the screening threshold, was |log_2_(FoldChange)| > 1 and *q* value <0.005. The results were performed by volcano plot, Venn diagram, heatmap, and clustering analysis.

### 2.9. DEGs Functional Analysis

GO (Gene Ontology, https://www.geneontology.org/) and KEGG (Kyoto Encyclopedia of Genes and Genomes, https://www.kegg.jp) were performed to analyse the function of DEGs between AS2.2174 and BM1403.

### 2.10. Statistical Analysis

Statistical analyses were carried out using the GraphPad Prism software, and the results were expressed as means and standard deviations (*x* ± *s*). Murine survival study was assessed by log-rank chi square test. Multiple groups were compared by one-way analysis of variance (ANOVA) followed by the Student–Newman–Keuls (SNK-q) test. Statistical significance was set at *P* < 0.05, and GO analysis statistical significance “corrected *P* value” was set at *P* < 0.05.

## 3. Results

### 3.1. Strains and BMDC Coincubation

**Figure 1:**
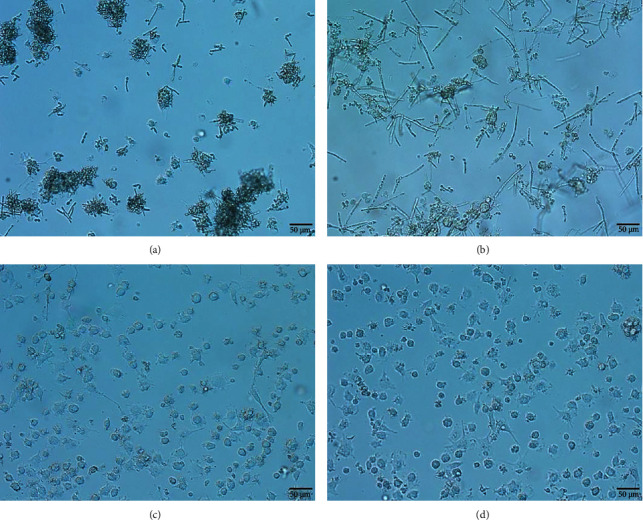
*T. asahii* strains and BMDC coincubation for 24 h. (a) Living BM1403 was adhered by BMDC. (b) Living AS2.2174 was adhered by BMDC. (c) Heat-inactivated BM1403 was mostly phagocytosed by BMDC. (d) Heat-inactivated AS2.2174 was mostly phagocytosed by BMDC.

**Figure 2:**
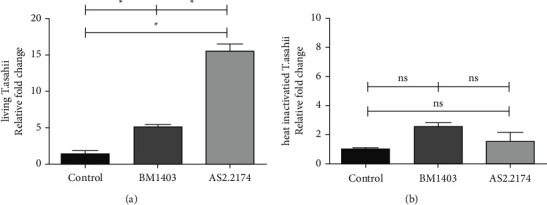
*T. asahii* induces the expression of TNF-*α* in BMDC for 24 h: (a) living *T. asahii* with BMDC; (b) heat-inactivated T. *asahii* with BMDC. (ns) *P* >  0.05 and (^*∗*^) *P* < 0.05.

After 24 h of coincubation, most of the living *T. asahii* strains were adhered by BMDC because of filamentation response and heat-inactivated *T. asahii* strains were mostly phagocytosed by BMDC (Figure 1). We selected TNF-*α* as the representative inflammatory response of BMDC to *T. asahii*; real-time PCR showed living BM1403 induced much lower expression of TNF-*α* by BMDC than living AS2.2174, and there were no differences when they were heat inactivated (Figure 2).

### 3.2. Murine Survival Study

**Figure 3:**
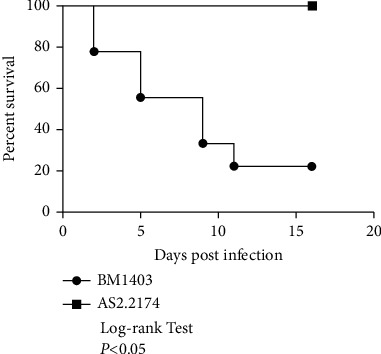
Survival curves of mice infected with *T. asahii* strains.

Experimental mice were inoculated intravenously with *T. asahii* strains for 16 consecutive days. Log-rank chi square test showed that mice infected with BM1403 performed significant higher survival rate than AS2.2174 (*P* < 0.05): the median survival time of mice infected with AS2.2174 was 9 days while all the mice infected with BM1403 survived till the end of the trial (Figure 3).

### 3.3. Expression Level Analysis

**Figure 4:**
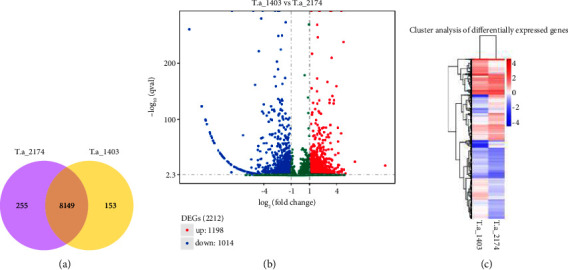
DEGs of AS2.2174 and BM1403. (a) Venn diagram. (b) Volcano plots. (c) Heatmap and clustering analysis of AS2.2174 and BM1403. (T. a_2174) AS2.2174, and (T. a_1403) BM1403.

We used HTSeq and DEGseq software to analyse the transcriptome profiles of *T. asahii* strains AS2.2174 and BM1403. A total of 8149 shared DEGs between the two strains were identified (Figure 4(a), Supplementary Table S1). Among these DEGs, 2212 were statistically significant according to the screening threshold previously described as |log_2_(FoldChange)| > 1 and *q* value<0.005, of which 1014 were downregulated and 1198 were upregulated in BM1403 compared to AS2.2174 (Figure 4(b), Supplementary Table S2, Supplementary Table S3). Heatmap and clustering analysis revealed significant changes in the mRNA expression profiles of the two strains (Figure 4(c)).

### 3.4. GO Enrichment Analysis

The GO enrichment analysis of DEGs was categorized as biological process (BP), molecular function (MF), and cellular component (CC). In the downregulated DEGs of BM1403 compared to AS2.2174, terms of cellular molecule metabolic process and nucleic acid metabolism were significantly enriched in BP (*P* < 0.05), such as regulation of cellular metabolic process (GO: 0031323), regulation of RNA metabolic process (GO: 0051252), and regulation of gene expression (GO: 0010468), and nucleic acid-binding transcription factor activity (GO: 0001071) and sequence-specific DNA-binding transcription factor activity (GO: 0003700) were significantly enriched in MF (*P* < 0.05, Table 1, Supplementary Table S4). In the upregulated DEGs, terms of DNA repair were enriched in BP, such as response to DNA damage stimulus (GO: 0006974), and terms of host cell were enriched in CC, such as host intracellular part (GO: 0033646), but were not significant (*P* > 0.05, Table 2, Supplementary Table S5).

### 3.5. KEGG Enrichment Analysis

KEGG enrichment analysis indicated that valine, leucine, and isoleucine degradation (cne: 00280) was the only one significantly enriched pathway in downregulated DEGs of BM1403 compared to AS2.2174 (*P* < 0.05, Table 3, Supplementary Table S6) and other pathways were mainly associated with amino acid and fatty acid metabolism, such as butanoate metabolism (cne: 00650), arginine and proline metabolism (cne: 00330), and fatty acid degradation (cne: 00071). In the upregulated DEGs, there were no significantly enriched pathways (*P* > 0.05, Table 4, Supplementary Table S7); most pathways were associated with nucleic acid and amino acid metabolism, gene replication and repair, such as DNA replication (cne: 03030), mismatch repair (cne: 03430), and tryptophan metabolism (cne: 00380).

## 4. Discussion

To survive inside the host during chronic infection, fungus must be able to adapt to adverse environment and microevolution could be the driving force of immune response and virulence change, such as the experiment of continuous coincubation of *Candida glabrata* with a murine macrophage cell line for over six months [12], which resulted in alteration of morphology with increased immunogenicity (TNF-*α*) and increased virulence (mouse infection model). Our results could also see significant changes in immunogenicity and virulence, but on the contrary, after a 15-year-period infection, the microevolved *T. asahii* strain BM1403 induced much lower expression of TNF-*α* by BMDC and had a much superior survival rate than the AS2.2174 strain, tending to “peacefully” coexist with the host. This difference might be because of the two strains of our study were isolated from a systemic disseminated trichosporonosis patient, which offered a much more complicated environment that thrived most vitro studies. Ormerod et al. [13] analysed two serial isolates obtained from a immunodeficiency patient suffered an initial and relapse episode of cryptococcal meningoencephalitis; consistent with our results, the microevolved isolate (F2) also exhibited reduced virulence such as capsule size and melanization at 37°C than the initial strains (F0) and was amplified by mice survival model, and whole-genome sequencing uncovered transcriptional regulatory gene mutants.

We performed transcriptome sequencing analysis of the two serial isolates and found adaptions by DEGs of *T. asahii* in systemic infected patient. Our data identified 8149 shared DEGs, and 2212 DEGs showed statistical significance in BM1403 compared to AS2.2174. Among these, the GO terms significantly enriched in downregulated transcription and metabolic process, and the KEGG pathway significantly enriched in downregulated valine, leucine, and isoleucine degradation. Valine, leucine, and isoleucine, also known as branched-chain amino acids (BCAAs), are synthesized by fungi for important components of proteins and secondary metabolites, especially ribosomal protein [14]; hence, the KEGG pathway was highly consistent with the GO enrichment. By reviewing the literature [15–18], we found that BCAA auxotrophs in human fungal pathogens have decreased pathogenicity and virulence, which might illustrate the reduced immunogenicity and virulence of BM1403. Highly coincidence with these results, our team previously performed proteome analysis of the two *T. asahii* strains and demonstrated that the most common homologs in the more abundant DEPs (differentially expressed proteins) were in reduced virulence category, followed by unaffected pathogenicity and loss of pathogenicity [19]. Furthermore, the enzyme in BCAA biosynthesis pathway is a promising new target for antifungal drug discovery, as humans do not synthesize BCAAs [15, 20].

There are several limitations in our study. Firstly, genes involved in branched-chain amino acids (BCAAs) metabolism for *T. asahii* remained unclear in our study and previous work. Secondly, pathway-specific transcriptional regulation of BCAAs biosynthesis needs to be confirmed in our future work. Thirdly, we also need to provide much more specific evidences for the relationship between BCAAs auxotrophs and decreased pathogenicity and virulence in *T. asahii*.

In summary, our study demonstrated that microevolution induced by chronic infection could induce changes in immunogenicity, virulence, and transcriptome, which might lead *T. asahii* to coexist with the host. Future studies could amplify our deduction, and a better understanding of the BCAA pathway could provide new ways to deal with chronic *T. asahii* systemic infection.

## Figures and Tables

**Table 1:** Top 20 most enriched GO terms of BM1403 vs. AS2.2174 (downregulated).

GO accession	Description	Term type	Corrected *P* value	DEG item
GO: 0001071	Nucleic acid-binding transcription factor activity	Molecular function	0.02335^*∗*^	39
GO: 0003700	Sequence-specific DNA-binding transcription factor activity	Molecular function	0.02335^*∗*^	39
GO: 0031323	Regulation of cellular metabolic process	Biological process	0.02335^*∗*^	63
GO: 0080090	Regulation of primary metabolic process	Biological process	0.02335^*∗*^	63
GO: 0019222	Regulation of metabolic process	Biological process	0.02335^*∗*^	63
GO: 0060255	Regulation of macromolecule metabolic process	Biological process	0.02335^*∗*^	63
GO: 0006355	Regulation of transcription and DNA-dependent	Biological process	0.02335^*∗*^	60
GO: 0051252	Regulation of RNA metabolic process	Biological process	0.02335^*∗*^	60
GO: 2001141	Regulation of RNA biosynthetic process	Biological process	0.02335^*∗*^	60
GO: 0009889	Regulation of biosynthetic process	Biological process	0.02335^*∗*^	61
GO: 0010556	Regulation of macromolecule biosynthetic process	Biological process	0.02335^*∗*^	61
GO: 0031326	Regulation of cellular biosynthetic process	Biological process	0.02335^*∗*^	61
GO: 2000112	Regulation of cellular macromolecule biosynthetic process	Biological process	0.02335^*∗*^	61
GO: 0006351	Transcription and DNA-dependent	Biological process	0.02335^*∗*^	67
GO: 0019219	Regulation of nucleobase-containing compound metabolic process	Biological process	0.02335^*∗*^	60
GO: 0051171	Regulation of nitrogen compound metabolic process	Biological process	0.02335^*∗*^	60
GO: 0010468	Regulation of gene expression	Biological process	0.02335^*∗*^	61
GO: 0032774	RNA biosynthetic process	Biological process	0.024632^*∗*^	67
GO: 0010467	Gene expression	Biological process	0.10147	113
GO: 0016070	RNA metabolic process	Biological process	0.11602	77

^*∗*^ *P* < 0.05.

**Table 2:** Top 20 most enriched GO terms of BM1403 vs. AS2.2174 (upregulated).

GO accession	Description	Term type	Corrected *P* value	DEG item
GO: 0006281	DNA repair	Biological process	0.79186	26
GO: 0006974	Response to DNA damage stimulus	Biological process	0.79186	26
GO: 0016667	Oxidoreductase activity, acting on a sulfur group of donors	Molecular function	0.79186	16
GO: 0030154	Cell differentiation	Biological process	0.79186	4
GO: 0048869	Cellular developmental process	Biological process	0.79186	4
GO: 0018995	Host	Cellular component	0.79186	8
GO: 0033643	Host cell part	Cellular component	0.79186	8
GO: 0033646	Host intracellular part	Cellular component	0.79186	8
GO: 0033647	Host intracellular organelle	Cellular component	0.79186	8
GO: 0033648	Host intracellular membrane-bounded organelle	Cellular component	0.79186	8
GO: 0042025	Host cell nucleus	Cellular component	0.79186	8
GO: 0043245	Extraorganismal space	Cellular component	0.79186	8
GO: 0043656	Intracellular region of host	Cellular component	0.79186	8
GO: 0043657	Host cell	Cellular component	0.79186	8
GO: 0044215	Other organism	Cellular component	0.79186	8
GO: 0044216	Other organism cell	Cellular component	0.79186	8
GO: 0044217	Other organism part	Cellular component	0.79186	8
GO: 0004089	Carbonate dehydratase activity	Molecular function	0.79186	3
GO: 0015976	Carbon utilization	Biological process	0.79186	3
GO: 0015036	Disulfide oxidoreductase activity	Molecular function	0.79186	15

**Table 3:** Top 20 most enriched KEGG downregulated pathways of BM1403 vs. AS2.2174.

ID	Term	Corrected *P* value	Input number	Background number
Cne: 00280	Valine, leucine, and isoleucine degradation	0.00580431^*∗*^	15	34
Cne: 00290	Valine, leucine, and isoleucine biosynthesis	0.053355169	7	11
Cne: 00650	Butanoate metabolism	0.080686696	9	22
Cne: 00072	Synthesis and degradation of ketone bodies	0.176946066	4	5
Cne: 00770	Pantothenate and CoA biosynthesis	0.176946066	6	14
Cne: 01230	Biosynthesis of amino acids	0.176946066	21	102
Cne: 01110	Biosynthesis of secondary metabolites	0.221257052	41	251
Cne: 03040	Spliceosome	0.221257052	18	89
Cne: 00620	Pyruvate metabolism	0.221257052	9	34
Cne: 00330	Arginine and proline metabolism	0.221257052	9	34
Cne: 01210	2-Oxocarboxylic acid metabolism	0.258341272	8	30
Cne: 00310	Lysine degradation	0.305346323	7	26
Cne: 03010	Ribosome	0.320239935	19	106
Cne: 00071	Fatty acid degradation	0.33918399	6	22
Cne: 00380	Tryptophan metabolism	0.36536438	7	29
Cne: 00020	Citrate cycle (TCA cycle)	0.391332945	6	24
Cne: 00010	Glycolysis/gluconeogenesis	0.391332945	8	37
Cne: 03022	Basal transcription factors	0.448334492	6	26
Cne: 00640	Propanoate metabolism	0.612280107	4	17
Cne: 00360	Phenylalanine metabolism	0.612280107	3	11

^*∗*^ *P* < 0.05.

**Table 4:** Top 20 most enriched KEGG upregulated pathways of BM1403 vs. AS2.2174.

ID	Term	Corrected *P* value	Input number	Background number
Cne: 03430	Mismatch repair	0.183673351	10	22
Cne: 03440	Homologous recombination	0.183673351	9	19
Cne: 03420	Nucleotide excision repair	0.183673351	13	36
Cne: 03450	Nonhomologous end-joining	0.183673351	6	9
Cne: 00380	Tryptophan metabolism	0.187880258	11	29
Cne: 03410	Base excision repair	0.328168471	8	20
Cne: 00650	Butanoate metabolism	0.391046326	8	22
Cne: 00430	Taurine and hypotaurine metabolism	0.391046326	4	7
Cne: 00460	Cyanoamino acid metabolism	0.391046326	4	7
Cne: 00450	Selenocompound metabolism	0.391046326	4	7
Cne: 01212	Fatty acid metabolism	0.490418613	7	21
Cne: 03030	DNA replication	0.490418613	9	31
Cne: 00040	Pentose and glucuronate interconversions	0.554740424	6	18
Cne: 00670	One carbon pool by folate	0.612732812	4	11
Cne: 00360	Phenylalanine metabolism	0.612732812	4	11
Cne: 00760	Nicotinate and nicotinamide metabolism	0.612732812	4	11
Cne: 00310	Lysine degradation	0.612732812	7	26
Cne: 00130	Ubiquinone and other terpenoid-quinone biosynthesis	0.612732812	3	7
Cne: 01040	Biosynthesis of unsaturated fatty acids	0.612732812	3	7
Cne: 00561	Glycerolipid metabolism	0.620926744	6	22

## Data Availability

The data that support the findings of this study are openly available in the Genome Sequence Archive (Genomics, Proteomics, and Bioinformatics 2021) in the National Genomics Data Center (Nucleic Acids Res 2022), China National Center for Bioinformation/Beijing Institute of Genomics, Chinese Academy of Sciences [21, 22] (GSA: CRA015321) that are publicly accessible at https://bigd.big.ac.cn/gsa/browse/CRA015321.

## Supplementary Materials

Supplementary Materials See Supplementary Tables S1–S7 in the Supplementary Materials for more GO terms and KEGG pathways.
